# Glucagonoma-induced acute heart failure

**DOI:** 10.1530/EDM-14-0061

**Published:** 2014-11-01

**Authors:** Kun Zhang, Lukas J Lehner, Damaris Praeger, Gert Baumann, Fabian Knebel, Marcus Quinkler, Torsten K Roepke

**Affiliations:** 1 Department of Cardiology and Angiology, Charité – Universitätsmedizin Berlin, Charitéplatz 1, 10115, Berlin, Germany; 2 Department of Nephrology, Charité – Universitätsmedizin Berlin, Charitéplatz 1, 10115, Berlin, Germany; 3 Department of Endocrinology, Charité – Universitätsmedizin Berlin, Charitéplatz 1, 10115, Berlin, Germany

## Abstract

Neuroendocrine tumours (NETs) represent a broad spectrum of tumours, of which the serotonin-producing carcinoid is the most common and has been shown to cause right ventricular heart failure. However, an association between heart failure and NETs other than carcinoid has not been established so far. In this case report, we describe a 51-year-old patient with a glucagon-producing NET of the pancreas who developed acute heart failure and even cardiogenic shock despite therapy. Heart failure eventually regressed after initialising i.v. treatment with the somatostatin analogue octreotide. Chromogranin A as a tumour marker was shown to be significantly elevated, and it decreased with clinical improvement of the patient. The effects of long-time stimulation of glucagon on the myocardium have not been studied yet; however, sarcoplasmic reticulum calcium leak can be discussed as a possible mechanism for glucagon-induced heart failure.

**Learning points:**

- Glucagonoma can be a cause for heart failure.
- i.v. infusion of octreotide can be successfully used to treat glucagonoma-induced acute heart failure.
- We suggest that cardiac function should be monitored in all NET patients.

## Background

Neuroendocrine tumours (NET) cover a broad spectrum of different hormone-secreting tumours of the neuroendocrine system. Among them, the serotonin-producing carcinoid occurs most commonly. It is known that progressed stage carcinoid can cause endocard fibrosis of the right heart leading to pulmonary valve stenosis and tricuspid valve insufficiency, subsequently leading to right ventricular heart failure (1). Overproduction of serotonin is proposed to be causal; however, the pathogenesis is still incompletely understood. NETs other than carcinoid have not been linked with heart disease yet.

Within the NETs, glucagon-producing tumours of the pancreas are fairly rare and can be associated with syndromes such as MEN1. Clinical manifestations of glucagonoma include necrolytic migratory erythema, loss of weight, local symptoms due to tumour growth, anaemia, diabetes mellitus, diarrhoea and thromboembolism (2). Localised glucagonoma without metastases show a good prognosis after surgical removal, whereas metastases imply a rather poor prognostic outcome (3).

In this case, we report on a patient with glucagonoma who presented with left ventricular heart failure and developed cardiogenic shock. Diagnostics and literature review led to the differential diagnosis of glucagonoma-induced heart failure. It is the second case described in the literature so far.

## Case presentation

We report on a 51-year-old female patient with MEN1 syndrome characterised by a heterozygous Q453X mutation in exon 10, which was diagnosed 5 years ago. Due to hyperparathyroidism and a pancreatic tumour, she underwent parathyroidectomy and pancreatic tail resection in the past. Histopathology of the pancreatic tumour revealed a glucagonoma with positive immunostaining for glucagon, chromogranin A and synaptophysin; negative immunostaining for gastrin, insulin, serotonin, somatostatin and pancreatic polypeptide. Moreover, the tumour showed the expression of somatostatin receptor type 2 (*SSTR2*), implicating responsiveness to somatostatin analogue therapy. As comorbidity, a multifactorial terminal kidney failure with haemodialysis three times a week has existed for almost 3 years. In the further course of disease, the glucagonoma relapsed with hepatic metastases, currently controlled as a stable disease under conservative treatment using everolimus 10 mg/day p.o. and octreotide long-acting release (LAR) 30 mg i.m. every 3 weeks.

In the present case, the patient came to the emergency room with dyspnoea and cough. Based on atypical bilateral infiltrations in the X-ray and highly elevated c-reactive protein (CRP), she was hospitalised with the diagnosis of pneumonia and treated with antibiotics while everolimus was paused; the last octreotide LAR injection was given 2 weeks prior to this. In the following days, her symptoms improved with normalising infection parameters. After 10 days of hospitalisation, however, her condition rapidly declined with progressive dyspnoea, orthopnoea and somnolence leading to admission to the intensive care unit.

On admission, the patient showed sinus tachycardia and hypotension. Echocardiography revealed acute heart failure with a severely impaired left ventricular ejection fraction (LVEF) of 10%. Heart disease was not known previously. Only moderate valvular dysfunction and no significant hypertrophy or dilatation was seen indicating a rather recent development of heart disease. Infusions with dobutamine and nitroprusside sodium were immediately started, but could not improve LVEF. Despite treatment, the patient developed cardiogenic shock, leading to intubation. By using PiCCO technology for haemodynamic measurements, we could confirm cardiogenic shock and clearly ruled out septic shock based on the following data: severely decreased cardiac index of 0.8 l/min per m^2^ (reference 3–5 l/min per m^2^), highly elevated systemic vascular resistance index of 3800 dyn×s×cm^−5^×m^2^ (reference 1700–2500 dyn×s×cm^−5^×m^2^).

## Investigation

Angiography ruled out coronary artery disease. Myocardial biopsy showed hypertrophy of cardiomyocytes and slight interstitial dilatation, but no signs of inflammation or metabolic heart disease. No cardiotoxic medication could be found in her previous medical history. Serum PCR for cardiotropic viral pathogens including parvovirus B19, coxsackie-A/B-virus, ECHO-virus, cytomegalovirus (CMV), Epstein-Barr virus (EBV), HHV-1, HHV-2, HHV-6, and HHV-8 was negative. No bacterial or fungal pathogen could be isolated from repeatedly collected samples of body fluids. At this point, the aetiology of heart failure remained unclear.

An association between glucagonoma and heart failure is not known. However, we found one case report by Chang-Chretien *et al*. (4) describing a 54-year-old female patient with glucagonoma-induced cardiomyopathy. This patient presented an LVEF of 15% with no valvular disease, clean coronary arteries, and in histopathology with only signs of myocardial hypertrophy and interstitial fibrosis – very similar to our patient. Surgical removal of the tumour led to complete normalisation of left ventricular function within 8 months. As pathomechanism, the authors suspected tachycardiomyopathy based on the chronotropic effect of glucagon.

## Treatment

Assuming that glucagonoma induced heart failure in our patient, we started therapy with continuous infusion of octreotide at a rate of 50 μg/h after an initial bolus of 50 μg, as surgery was not an option. In the following days, left ventricular (LV) function gradually improved, catecholamine treatment could be ended and the patient was extubated under stable haemodynamic conditions. Within 3 weeks, LVEF recovered up to 45% with a cardiac index of 3.7 l/min per m^2^. We determined the levels of chromogranin A, which is used as biomarker in NETs and correlates with hormone secretion (5). The levels of chromogranin A were at 1100 μg/l (reference 19–150 μg/l) under stable disease 1 year ago. Intriguingly, it was significantly increased to 4174 μg/l at the point of acute heart failure, and it markedly decreased after 1 day (1918 μg/l) and was back to baseline after 3 weeks (1223 μg/l) upon continuous octreotide treatment. Glucagon levels could not be determined due to the lack of a reliable test.

## Discussion

The pathomechanism of glucagon-induced heart failure remains subject to discussion. Glucagon binds to the glucagon receptor in the plasma membrane and leads to intracellular increase in cAMP with positive inotropic and chronotropic effects. This effect is known to be independent of β adrenergic receptors (6). Glucagon has been studied in the treatment of heart failure in the 1970s (7). Yet, it soon became obsolete due to its lacking impact on chronic heart failure and the frequent occurrence of gastrointestinal complications limiting its usefulness (8). Hence, data on long-time effects of glucagon on the myocardium are missing. As cAMP activates protein kinase A leading to phosphorylation of plasmalemmal L-type calcium channels and therefore increasing calcium influx, which in turn triggers release of calcium from intracellular stores, we believe that long-term glucagon stimulation of cardiomyocytes may cause sarcoplasmic reticulum calcium leak, leading to the activation of hypertrophy and heart failure signalling pathways (9) (10). Of note, myocardial biopsy of our patient and of the patient described by Chang-Chretien *et al*. revealed hypertrophy of myocytes.

In a retrospective view, we assume that congestive heart failure started to develop in the course of primary disease. Infection could have been a trigger for acute decompensation, possibly aggravated by pausing antiproliferative therapy with everolimus and simultaneously enhanced demand for octreotide, which may have led to excessive tumour activity with overproduction of glucagon. The trend of chromogranin A levels is indicative, but is not proving, because chromogranin A is a general product of endocrine secretion. Limiting factors in the assessment of chromogranin A in our patient are the use of proton pump inhibitors and terminal kidney failure with haemodialysis. However, both factors have been present years in advance and cannot explain the significant relative increase in chromogranin A at the point of acute heart failure. Considering the results of the initial immunostaining, we can assume that the tumour is indeed responsive to octreotide, and it seems unlikely that other hormones were causative for heart failure in this patient.

This case report demonstrates that glucagonoma can be a cause for acute heart failure, which moreover is reversible under continuous therapy with octreotide. It supports the idea that there is a link between NET and heart failure other than carcinoid heart disease. Therefore, it seems feasible to suggest that cardiac function should be monitored in a routine manner in all NET patients and under specific circumstances even in closer intervals. However, further studies are needed to elucidate the underlying pathomechanisms.

## Patient consent

We received consent from the husband of the patient.

## Author contribution statement

K Zhang was responsible for writing the manuscript. L J Lehner, D Praeger, G Baumann, F Knebel, M Quinkler and T K Roepke reviewed and edited the manuscript. All authors were involved in the treatment of the patient.
